# Institutional Needs

**Published:** 1915-03-27

**Authors:** 


### Institutional Needs.

### FELLOW'S MEDICAL MANUFACTURING COMPANY.

Under the title of "Memoranda for Busy Physicians" the Fellow's Medical Manufacturing Company, of New York, has issued a pamphlet to the medical profession of this country. It aims at incorporating in brief form "some of the more recent diagnostic knowledge not yet completely diffused" on a variety of points for the benefit of medical men engaged in private practice. Were it not that the Insurance Act has produced an apparent demand for such brief *résumés* of knowledge, which can only be really acquired by intimate study, the natural view would be that these details were subservient to the particulars of Fellow's compound syrup of hypophosphites contained at the end. That, however, needs no recommendation beyond what its reputation has achieved

### "LYSOL" EVANS

As a valuable germicide for disinfecting purposes "Lysol" Evans, manufactured by Evans, Sons, Lescher, and Webb, Limited, commands and is receiving attention. The makers claim it as a carefully prepared all-British product which is the nearest possible approach to the original "Lysol," and they are making special arrangements to ensure prompt delivery for orders, which was not an easy matter a little while ago. As a germicide, as a disinfectant for sinks and drains, and for general purposes "Lysol" Evans is obtainable in one-gallon tins at 11s. each, as well as in smaller quantities from 4 to 32 oz. Now that the tendency appears to be to prefer schools and other buildings for hospitals instead of utilising existing infirmary accommodation or building new temporary hospitals on vacant sites, the need is redoubled for careful disinfection of all premises. For such a purpose the requirements are a powerful British-made germicide equal in value to the former foreign products, and the claim of "Lysol" Evans to approximate to this ideal has much to commend it.

